# Monopulse Secondary Surveillance Radar Coverage—Determinant Factors

**DOI:** 10.3390/s21124198

**Published:** 2021-06-18

**Authors:** Gheorghe Minteuan, Tudor Palade, Emanuel Puschita, Paul Dolea, Andra Pastrav

**Affiliations:** 1 Communications Department, Technical University of Cluj-Napoca, 28 Memorandumului Street, 400114 Cluj-Napoca, Romania; Gheorghe.Minteuan@com.utcluj.ro (G.M.); Tudor.Palade@com.utcluj.ro (T.P.); Emanuel.Puschita@com.utcluj.ro (E.P.); Paul.Dolea@com.utcluj.ro (P.D.); 2 Department of Communication, Navigation and Surveillance, Romanian Air Traffic Services Administration, 1 Dambovitei Street, 400584 Cluj-Napoca, Romania

**Keywords:** MSSR, MSSR coverage, horizontal factor, vertical factor, radiation pattern lobing, SUM beam

## Abstract

This paper presents a comprehensive study on monopulse secondary surveillance radar (MSSR) coverage. The design and radiation pattern of an improved MSSR antenna is presented herein, highlighting the horizontal and vertical factors of the SUM beam. Moreover, the impact of other determinant factors, such as signal reflection and atmospheric refraction, on the radar coverage were assessed in this work. Real positioning measurement data and coverage simulations were used to support and exemplify theoretical findings.

## 1. Introduction

Monopulse Secondary Surveillance Radar (MSSR) systems rely on two key aspects for accurate aircraft (AVO) detection (i.e., decoding the AVO identification code) and positioning (i.e., determining the slant distance, elevation, and azimuth of the AVO). First, for detection, the AVO must be equipped with a transponder that generates trains of pulses in response to interrogation from ground equipment. Second, for correct positioning, the monopulse radar must be able to process the received pulses and determine, with improved precision, the AVO azimuth. To this extent, the MSSR must employ special antennas with multiple radiant elements, fed with rigorously determined phases and signal levels to ensure strong positioning performance and optimum coverage.

However, the ability of MSSR systems to identify and locate a given aircraft is greatly impacted by other factors, including antenna radiation pattern, transmitting power, receiver sensitivity, MSSR antenna placement (location relief), Earth curvature, atmospheric refraction, external noise, and multipath propagation, among others. [1].

Several books were published in the 1980s and 1990s that presented the fundamentals of MSSR equipment [2,3,4], including operating principles, design and block diagrams of the equipment, coverage (including key aspects such as atmospheric refraction, line-of-sight (LoS) propagation, lobing due to ground reflections), and the radiation pattern of the required SUM, DIFF and OMNI antennas. Although these works included valuable information about antennas and antenna arrays, a detailed design of the MSSR antenna were not provided.

The International Civil Aviation Organization (ICAO) established the MSSR antenna requirements in [5] and [6], and several papers focused solely on specific aspects of antenna design, analyzing the radiation patterns obtained for various geometries [7,8,9,10,11]. The work in [7] proposed an MSSR antenna design containing 35 columns of dipoles. The phases and amplitudes of the feeding currents were determined using the Taylor method. The study focused only on the horizontal radiation pattern, showcasing a narrow main lobe of 2.4° and a gain of 27 dB.

Improvements for existing radar systems were presented in [12,13], highlighting the geometry of the antennas and the resulting radiation patterns. 

The work in [12] showed the modernization of RSP-10 radar systems. The proposed antenna unit design used a double-curved reflector and three independent feeds that were positioned vertically close to the reflector focus. The radiation pattern was depicted, showcasing a main lobe that was narrow horizontally and wide vertically. The gain was 28.62 dBi and the horizontal half-power beamwidth (HPBW) was approximately 3°. The authors state that various constraints generated the need for compromise, showing that a perfect solution may not exist.

The work in [13] presented upgrades for the Russian RSP-10 radar, combining the ICAO secondary radar system, the primary surveillance radar antenna at L band, ICAO and Russian UVD standard. The proposed design made use of a phased antenna array and common reflector to generate the SUM and DIFF patterns.

A representation of the surveillance volume of a single radar was depicted in [14]. The work highlighted the cone of silence (CoS)—the airspace above the radar that is invisible to the radar.

Only a few studies provided a more comprehensive view of radar coverage [15,16]. 

The work in [15] presented an algorithm that estimated the 3D coverage of a primary surveillance radar (PSR). The algorithm included aspects related to atmospheric refraction, Earth curvature, and ground reflection. The model was based on parabolic equations. An antenna with a sine cardinal (sinc) pattern was used in the simulation and the results showed that reflections could generate radar-blind zones. 

The MOSTDONT project [16] aimed at identifying and investigating potential certification issues for Mode S transponders in high density operational environments. The study focused on the performance of existing MSSR equipment, highlighting a number of issues associated with operating Mode S transponders, and providing useful insights concerning the impact of ground-induced multipath signal propagation. The report showed that, in practice, the lower boundary of a large vertical aperture (LVA) antenna was different from the theoretical (computed) pattern because “there are no pure plane wave and far-field conditions at low elevation angles close to the horizon” [16]. As such, a complete theoretical description could not be achieved, but the signal degradation could be assessed by means of measurement campaigns. 

In this context, the goal of this paper was to provide a thorough overview of MSSR coverage. First, an MSSR antenna was proposed. Although the proposed antenna design is similar to existing MSSR antennas, the proposed array geometry—along with the carefully determined feeding signals—led to an enhanced radiation pattern, more suitable for air traffic surveillance. The detailed theoretical background focused on the SUM beam and was presented along with the resulting radiation patterns. Moreover, the impact of critical aspects, such as atmospheric refraction and relief-generated reflections, were also taken into account when evaluating the MSSR coverage. The theoretical findings were then exemplified by means of measurements and real positioning data analyses, and through coverage simulations in Radio Mobile.

This paper is an extended version of the paper “MSSR Antenna Theory” [17] presented at the International Symposium on Electronics and Telecommunications ISETC 2020. This study developed a more comprehensive radar coverage assessment, including the following contributions:

- Proposal of an MSSR antenna with custom geometry and feeding signals for an improved radiation pattern.
- Determination of the SUM beam radiation pattern, illustrating the horizontal, vertical, and 3D characteristics of the proposed MSSR antenna.
- Assessment of reflection and refraction events in radar coverage, highlighting the cause for the lobing phenomenon.
- Evaluation of the impact of the curvature of the Earth on the MSSR range, uniquely illustrating the perceived flight angle variation with the slant distance for an AVO in flight at constant level.
- Estimation of the link budget based on the features of the proposed MSSR antenna.
- Illustration of the theoretical findings by means of real MSSR measurements and positioning and analysis of extracted positioning data.
- MSSR coverage simulation in Radio Mobile.

The remainder of this paper is organized as follows. Section 2 describes the proposed MSSR antenna and determines the radiation pattern (horizontal, vertical and 3D). Section 3 addresses important aspects with respect to radar coverage, such as reflection and refraction. Section 4 illustrates some of the theoretical findings by means of real positioning data analysis and evaluates the proposed antenna through simulations in Radio Mobile. Finally, Section 5 concludes the paper.

## 2. Proposed MSSR Antenna

**Figure 1:**
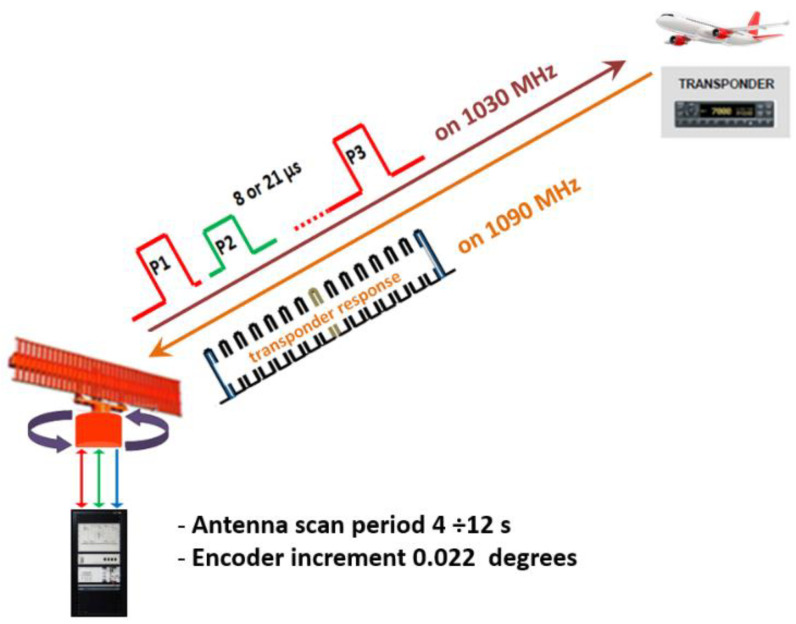
Classic MSSR–aircraft transponder dialogue.

MSSR is used for the detection and positioning in space of aircrafts equipped with transponders. During operation, the MSSR antenna performs continuous 360° rotational movements scanning for aircrafts and initiates a dialogue by issuing queries on the frequency 1030 MHz. According to [5,18], an aircraft within the MSSR main beam field of view (FoV) responds to the specific requests on the frequency 1090 MHz. The response of the aircraft transponder consists of 12 pulses of 450 ns (position modulated) inside the framing pulses. Figure 1 illustrates this dialogue. During rotations, the angular position of the MSSR antenna is known by the MSSR extractor due to the existing encoder. The MSSR-aircraft slant distance is determined based on time of flight, knowing the MSSR interrogation time, the transponder response reception time, and the transponder response delay (i.e., 3 μs for A-C dialogue). The decoded response provides the aircraft identifier (i.e., Mode A code when the P1–P3 interrogation pulse spacing is 8 μs) or the aircraft flight level (when the 21 μs P1–P3 interrogation pulse spacing is used). 

The ICAO requirements regarding the MSSR antenna [5,6] resulted in an MSSR antenna [2] combining three beams: SUM, CONTROL and DIFFERENCE.

The SUM beam must be narrow horizontally for coverage of 256 NM [19] and wide vertically to allow aircraft detection in the upper vertical half-plane, resulting in an LVA. 

**Figure 2:**
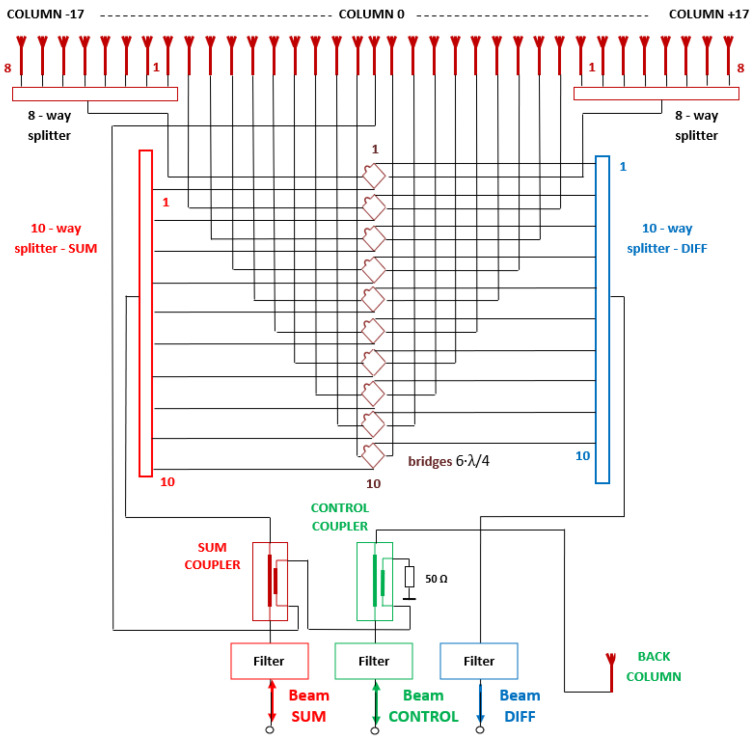
Block diagram of the proposed MSSR antenna.

Figure 2 shows the block diagram of the proposed MSSR antenna, highlighting the input/output for the SUM and CONTROL beams and the output for the DIFFERENCE beam. The antenna employs 35 vertical front columns and one vertical back column. The resulting SUM, CONTROL and DIFFERENCE beams depend on how the 36 vertical columns are connected through the 8- and 10-way splitters, 6·λ/4 bridges, and couplers—and on the electrical lengths of the feeders. In brief:

- the SUM beam is obtained by employing the 35 front columns of the antenna and, to a small extent, the back column. It is used for the MSSR-aircraft dialogue.
- the CONTROL beam is obtained by employing the back column and, through the CONTROL coupler (3 dB attenuation), the 35 front columns (with signal phase reversal). It is used for the validation of the MSSR request in the aircraft transponder and for the validation of the aircraft answer in the MSSR extractor.
- the DIFFERENCE beam is the sum of the signal differences of the 17 right-left pairs of front columns for the purpose of off boresight angle (OBA) evaluation in the radar extractor [3]. It is used to improve the extracted AVO azimuth.

**Figure 3:**
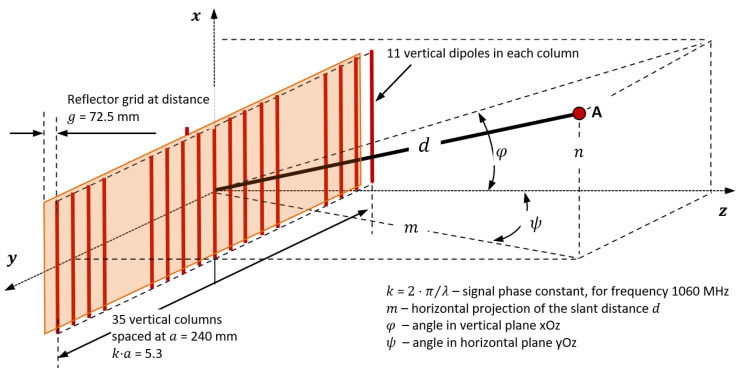
Geometry of proposed MSSR antenna.

Figure 3 shows the geometry of the proposed antenna (similar to the antennas presented in [20]), highlighting the elements used in the mathematical calculation.

The terms horizontal factor (OF) and vertical factor (VF) are used to determine the horizontal and vertical patterns of the SUM beam. The horizontal pattern is obtained computing |OF·OD|^2^, where OD is the dipole factor in the horizontal plane. Similarly, the vertical pattern is obtained computing |VF·VD|^2^, where VD is the dipole factor in the vertical plane. Through division by their maximum values, the SUM beam radiation patterns are converted into gains in the horizontal (G_horizontal (dB)_) and vertical (G_vertical (dB)_) planes. The SUM beam patterns are computed for 1060 MHz (the average between transmission and reception frequencies). The results of the theoretical SUM beam patterns’ calculation are comparable to those in [6,7,8,9,10,11].

### 2.1. The Dipole with Reflector

**Figure 4:**
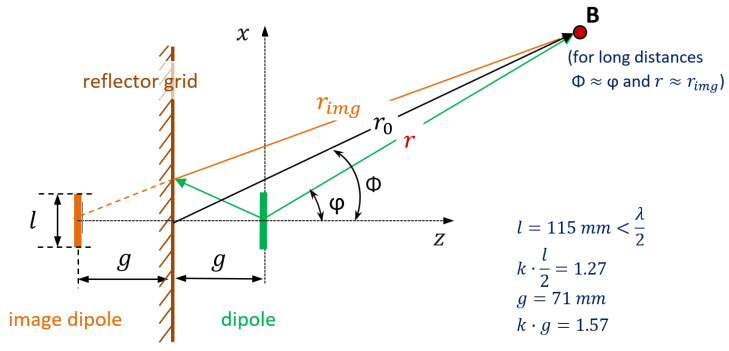
Dipole and image dipole due to the reflector grid.

Figure 4 illustrates the vertical dipole, accompanied by the reflector grid, considered in calculation of radiation. According to [21], the intensity of the electric field in point B for the dipole fed with the current *I*_0_ was (1), and for the image dipole (whose phase was reversed by reflection) was (2).

(1) $$E_{dipole\left(\varphi \right)}=\frac{j\cdot Z_{0}\cdot I_{0}\cdot e^{-j\cdot k_{t}\cdot r}}{2\cdot \pi \cdot r}\cdot \frac{\cos\left(k\cdot \frac{l}{2}\cdot \sin\varphi \right)-\cos\left(k\cdot \frac{l}{2}\right)}{\cos\varphi }$$

(2) $$E_{imgdipole\left(\varphi \right)}=-\frac{j\cdot Z_{0}\cdot I_{0}\cdot e^{-j\cdot k\cdot r_{img}}}{2\cdot \pi \cdot r_{img}}\cdot \frac{\cos\left(k\cdot \frac{l}{2}\cdot \sin\varphi \right)-\cos\left(k\cdot \frac{l}{2}\right)}{\cos\varphi }$$

The essential consideration in deducing the expressions (3) and (4) below is the vertical polarization of the electrical signal emitted/received by the vertical dipole.

The absolute value of (1) and (2) represents the magnitude which, related to its maximum, leads to VD in (3):

(3) $$VD_{\left(\varphi \right)}=\frac{\left[\cos\left(k\cdot \frac{l}{2}\cdot \sin\varphi \right)-\cos\left(k\cdot \frac{l}{2}\right)\right]\cdot \sin\left(k\cdot g\cdot \cos\varphi \right)}{\cos\varphi \cdot \left[1-\cos\left(k\cdot \frac{l}{2}\right)\right]\cdot \sin\left(k\cdot g\right)}$$

The OD determination is based on the constant circular pattern of a dipole without reflector, resulting in (4).

(4) $$OD_{\left(\psi \right)}=\sin\left(k\cdot g\cdot \cos\psi \right)/\sin\left(k\cdot g\right)$$

### 2.2. Horizontal Pattern of SUM Beam

#### 2.2.1. Currents Required for Feeding the Columns

As presented in [22], the Taylor distribution of the currents *I_(y)_* injected in the columns for the SUM beam of the antenna sketched in Figure 1 is (5) (similar to [7] and [23]).

(5) $$I_{\left(y\right)}=1+2\cdot \overset{\bar{n}-1}{\underset{p=1}{\sum }}\left[SF_{\left(p,\bar{n}\right)}\cdot \cos\left(p\cdot \frac{\pi \cdot y}{17}\right)\right]$$

where the coefficient $SF_{\left(p,\bar{n}\right)}$ is:

(6) $$SF_{\left(p,\bar{n}\right)}=\frac{{\left(\left(\bar{n}-1\right)!\right)}^{2}}{\left(\bar{n}-1+p\right)!\cdot \left(\bar{n}-1-p\right)!}\cdot \overset{\bar{n}-1}{\underset{m=1}{\prod }}\left(1-{\left(\frac{y}{u_{m}}\right)}^{2}\right)$$

and $u_{m}$ is:

(7) $$u_{m}=\pm \sigma \cdot \sqrt{A^{2}+{\left(m-1/2\right)}^{2}}\;\mathrm{for}\;1\leq m\leq n$$

where *n* = 17 represents the number of side columns and

(8) $$\sigma =\bar{n}/\sqrt{A^{2}+{\left(\bar{n}-1/2\right)}^{2}}$$

The constant A is given in (9) and imposed so that the ratio between the maximum secondary lobe and the central lobe is R. If R is in dB, it must be converted into a voltage ratio.

(9) cosh(π·A)=R

Computing the currents applied to the vertical columns was laborious, but the terms $SF_{\left(q,\bar{n}\right)}$ became null for q ≥ 10 due to the terms of the form (9 − q)! = ±∞ in the denominator.

For a ratio of −35 dB between the maximum secondary lobe amplitude and the central lobe, converted into a constant voltage ratio R = 56.23, and according to (9), results A = 1.5032.

Here, $\bar{n}=10$ is chosen arbitrarily, the condition being to obtain a narrow beamwidth of the SUM beam (in ICAO documents [5,6] the beamwidth at 3 dB attenuation is 2.5°). As such:

(10) $$\sigma =10/\sqrt{1.5032^{2}+{\left(10-1/2\right)}^{2}}=1.04$$

(11) $$u_{m}=\pm 1.04\cdot \sqrt{2.259+{\left(m-1/2\right)}^{2}}$$

(12) $$SF_{\left(p,10\right)}=\frac{{\left(9!\right)}^{2}}{\left(9+p\right)!\cdot \left(9-p\right)!}\cdot \overset{9}{\underset{m=1}{\prod }}\left(1-\frac{p^{2}}{1.04\cdot \left(2.25+{\left(m-1/2\right)}^{2}\right)}\right)$$

For example, the current applied to the 8th column, *I*_(7)_, is given in (13), while *SF*_(7,10)_ is expressed in (14).

(13) $$I_{\left(7\right)}=1+2\cdot \overset{\bar{n}-1}{\underset{p=1}{\sum }}SF_{\left(p,\bar{n}\right)}\cdot \cos\left(p\cdot \frac{\pi \cdot 7}{17}\right)$$

(14) $$SF_{\left(7,10\right)}=\frac{{\left(9!\right)}^{2}}{16!\cdot 2!}\cdot \overset{9}{\underset{m=1}{\prod }}\left(1-{\left(7/u_{m}\right)}^{2}\right)$$

Table 1 summarizes the currents to be applied to the 35 vertical columns for the SUM beam.

#### 2.2.2. The Horizontal Factor

To obtain the LVA pattern of the SUM beam, we proposed a phase difference (*ψ_y_*) between the phase of the central column and the phase of columns ± y. For the reception in A (Figure 3), horizontally, the expression of the electric field *EO_(ψ)_* (relative to the maximum for *ψ* = 0) as a function of the effective currents *I_ef(y)_* applied to the columns was written in (15), using Euler’s formula as in [21,22,24,25,26].

(15) $$EO_{\left(\psi \right)}=I_{0}+\overset{17}{\underset{n=1}{\sum }}\left[I_{ef\left(n\right)}\cdot e^{-j\cdot \left(n\cdot k\cdot a\cdot \sin\psi -\psi_{n}\right)}+I_{ef\left(n\right)}\cdot e^{+j\cdot \left(n\cdot k\cdot a\cdot \sin\psi +\psi_{n}\right)}\right]\;\;=I_{0}\;+\overset{17}{\underset{n=1}{\sum }}[I_{ef\left(n\right)}\cdot e^{+j\cdot \psi_{n}}\cdot \left(e^{+j\cdot (n\cdot k\cdot a\cdot \sin\psi )}+e^{-j\cdot \left(n\cdot k\cdot a\cdot \sin\psi \right)})\right]\;\;=I_{0}+2\cdot \overset{17}{\underset{n=1}{\sum }}[I_{ef\left(n\right)}\cdot \cos\left(n\cdot k\cdot a\cdot \sin\psi \right)\cdot e^{+j\cdot \psi_{n}}]$$

The absolute value of (15) is the magnitude of the electric field intensity in point A horizontally, resulting in OF as in (16). The phase difference introduced to the pairs of symmetrical columns *y* affects their amplitude terms by the factor *cosψ_y_*.

(16) $$OF_{\left(\psi \right)}=\left|EO_{\left(\psi \right)}\right|=I_{0}+2\cdot I_{1}\cdot \cos\psi_{1}\cdot \cos\left(1\cdot k\cdot a\cdot \sin\psi \right)+\ldots +2\cdot I_{17}\;\cdot \cos\psi_{17}\cdot \cos\left(17\cdot k\cdot a\cdot \sin\psi \right)$$

According to (17), each column current *I_(y)_* depends on the effective currents *I_ef(y)_* through cos*ψ_y_*.

(17) $$I_{\left(y\right)}=I_{ef\left(y\right)}\cdot \cos\psi_{y}$$

**Figure 5:**
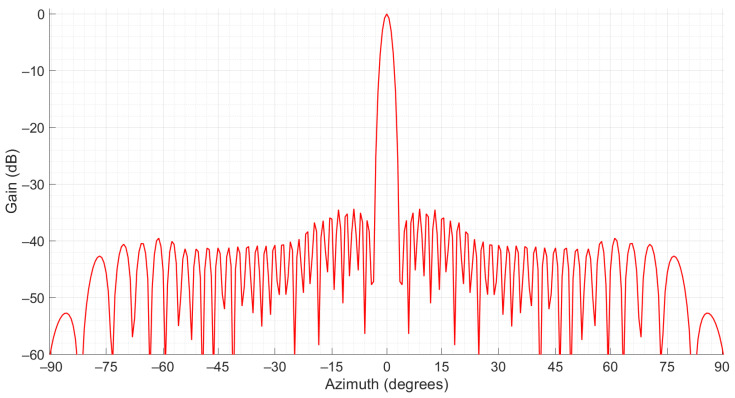
Horizontal gain of the SUM beam on 1060 MHz.

The horizontal pattern |OF·OD|^2^ was represented as the horizontal gain of the SUM beam relative to the maximum value for *ψ* = 0, denoted *G_horizontal(ψ)(dB)_*, expressed in (18) and illustrated in Figure 5 where all the columns were fed in phase.

(18) $$\begin{array}{cc} G_{horizontal\left(\psi )(dB\right)}= & 20\\ & \cdot \log|\sin\left(1.57\cdot \cos\psi \right)\cdot (1.64+2\cdot 1.63\\ & \cdot \cos\left(1\cdot 5.2\cdot \sin\psi \right)+2\cdot 1.60\\ & \cdot \cos\left(2\cdot 5.2\cdot \sin\psi \right)+2\cdot 1.55\cdot \cos\left(3\cdot 5.2\cdot \sin\psi \right)+2\cdot 1.48\cdot \cos\left(4\cdot 5.2\cdot \sin\psi \right)+2\cdot 1.4\\ & \cdot \cos\left(5\cdot 5.2\cdot \sin\psi \right)+2\cdot 1.31\cdot \cos\left(6\cdot 5.2\cdot \sin\psi \right)+2\cdot 1.2\cdot \cos\left(7\cdot 5.2\cdot \sin\psi \right)+2\cdot 1.08\\ & \cdot \cos\left(8\cdot 5.2\cdot \sin\psi \right)+2\cdot 0.97\cdot \cos\left(9\cdot 5.2\cdot \sin\psi \right)+2\cdot 0.85\cdot \cos\left(10\cdot 5.2\cdot \sin\psi \right)+2\cdot 0.73\\ & \cdot \cos\left(11\cdot 5.2\cdot \sin\psi \right)+2\cdot 0.62\cdot \cos\left(12\cdot 5.2\cdot \sin\psi \right)+2\cdot 0.51\cdot \cos\left(13\cdot 5.2\cdot \sin\psi \right)+2\cdot 0.40\\ & \cdot \cos\left(14\cdot 5.2\cdot \sin\psi \right)+2\cdot 0.34\cdot \cos\left(15\cdot 5.2\cdot \sin\psi \right)+2\cdot 0.33\cdot \cos\left(16\cdot 5.2\cdot \sin\psi \right)+2\cdot 0.33\\ & \cdot \cos\left(17\cdot 5.2\cdot \sin\psi \right)|-20\cdot \text{log}34.4 \end{array}$$

As Figure 5 shows, the horizontal pattern of the SUM beam contained a narrow main lobe. The HPBW was approximately 2.41° and the side lobe level (SLL) was −34 dB. These values showed good compliance with the recommendations in [18,27] that stated that the HPBW should fall within 2–3° (with a typical good value of 2.4°) with an SLL of at least −24 dB. 

### 2.3. Vertical Pattern of Sum Beam

#### 2.3.1. Column Factor

**Figure 6:**
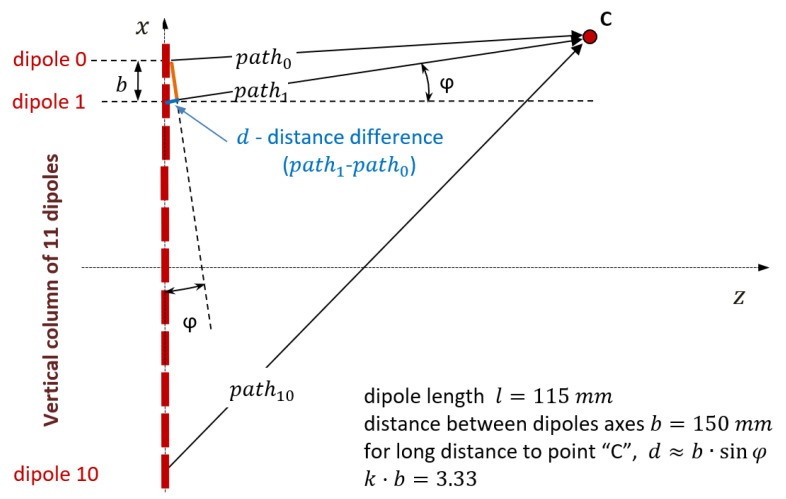
MSSR dipole column.

Figure 6 shows a column of the MSSR antenna and Table 2 presents the distribution of the 11 dipole currents, I_(dx)_, and their phase differences, φ_x_, relative to dipole 5. All the columns were identical, being fed in phase with the proper currents (as computed above).

The currents and phases of the dipole elements in a column were determined using the MATLAB Phased array design toolbox v2.5 [28]. This toolbox allows the user to provide as input a desired pattern and extract as output the estimated currents and phases required to generate such a pattern. The extracted currents and phases were synthesized as shown in Table 2. Next, these values were used for the calculation of the Column Factor (CF).

CF was introduced as the vertical factor for the column y, in the vertical plane perpendicular to the antenna. CF_(φ)_ = |EC_(φ)_|, where EC_(φ)_ in (19) represents the relative electric field (to its maximum for φ = 0) of the column y in point “C”:

(19) $${\mathrm{EC}}_{\left(\varphi \right)}=\overset{10}{\underset{x=0}{\sum }}\left(I_{\left(\mathrm{dx}\right)}\cdot e^{-j\cdot \left(x\cdot k\cdot b\cdot \sin\varphi +\varphi_{x}\right)}\right)$$

Considering dipole 5 as the reference, expression (19) is rewritten as (20):

(20) $$\begin{array}{cc} CF_{\left(\varphi \right)}=I_{d5}+I_{d4} & \cdot (e^{+j\cdot \left(1\cdot k\cdot b\cdot \sin\varphi +\varphi_{4}\right)}+e^{-j\cdot \left(1\cdot k\cdot b\cdot \sin\varphi +\varphi_{4}\right)})+I_{d3}\cdot (e^{+j\cdot \left(2\cdot k\cdot b\cdot \sin\varphi +\varphi_{3}\right)}\\ & +e^{-j\cdot \left(2\cdot k\cdot b\cdot \sin\varphi +\varphi_{3}\right)})+\ldots +I_{d0}\cdot (e^{+j\cdot \left(5\cdot k\cdot b\cdot \sin\varphi +\varphi_{0}\right)}+e^{-j\cdot \left(5\cdot k\cdot b\cdot \sin\varphi +\varphi_{0}\right)})\\ & =I_{d5}+2\cdot I_{d4}\cdot \cos\left(1\cdot k\cdot b\cdot \sin\varphi +\varphi_{4}\right)+\ldots +2\cdot I_{d0}\cdot \cos\left(5\cdot k\cdot b\cdot \sin\varphi +\varphi_{0}\right) \end{array}$$

The resulting CF is expressed in (21).

(21) $$\begin{array}{cc} CF_{\left(y,\varphi \right)}=1+2\cdot & 0.73\cdot \cos\left(1\cdot 3.33\cdot \sin\varphi +0.86\right)+2\cdot 0.39\cdot \cos\left(2\cdot 3.33\cdot \sin\varphi +1.14\right)+2\cdot 0.36\\ & \cdot \cos\left(3\cdot 3.33\cdot \sin\varphi +1.34\right)+2\cdot 0.2\cdot \cos\left(4\cdot 3.33\cdot \sin\varphi +1.82\right)+2\cdot 0.13\\ & \cdot \cos\left(5\cdot 3.33\cdot \sin\varphi +1.47\right) \end{array}$$

#### 2.3.2. Vertical Factor

The Vertical Factor (VF) for the SUM beam was obtained by summing the CF for the 35 vertical columns (columns fed with proper currents I_(y)_). VF was expressed in (22).

(22) $${VF}_{\varphi }=(I_{0}+2\cdot I_{1}+\ldots +2\cdot I_{17})\cdot {CF}_{\left(\varphi \right)}$$

**Figure 7:**
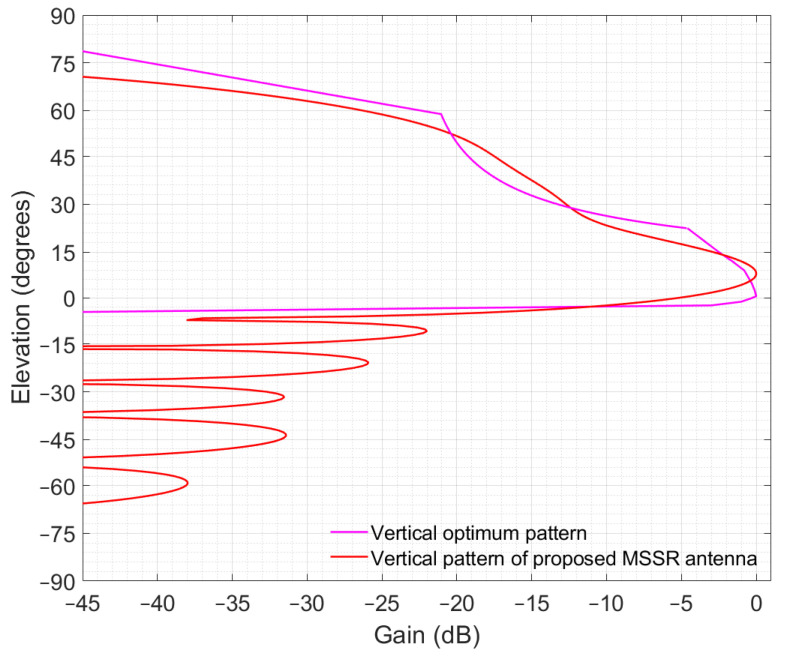
Vertical gain of the SUM beam on 1060 MHz.

The vertical pattern |VF·VD|^2^ was written as gain (relative to the maximum for *φ* = 0) in (23) with illustration in Figure 7.

(23) $$\begin{array}{cc} G_{vertical\left(\varphi \right)\left(dB\right)}= & 20\\ & \cdot \log|((\cos\left(1.27\cdot \sin\varphi -0.29\right)\cdot \sin(1.57\cdot \cos\varphi )/\cos\varphi )\cdot (1+2\cdot 0.73\\ & \cdot \cos\left(1\cdot 3.33\cdot \sin\varphi +0.86\right)+2\cdot 0.39\cdot \cos\left(2\cdot 3.33\sin\varphi +1.14\right)+2\cdot 0.36\\ & \cdot \cos\left(3\cdot 3.33\cdot \sin\varphi +1.34\right)+2\cdot 0.2\cdot \cos\left(4\cdot 3.33\sin\varphi +1.82\right)+2\cdot 0.13\\ & \cdot \cos\left(5\cdot 3.33\cdot \sin\varphi +1.47))\right|-20\cdot \log3.1 \end{array}$$

A phase shift, *ψ_y_*, can be introduced for column pairs *±y* affecting OF by the term cos*ψ_y_*, according to relation (17) and the CF by the term $e^{+j\psi_{y}}$, according to (20). Consequently, VF will be affected, being described by a more complicated mathematical expression. However, for the proposed antenna, all the columns were fed in phase.

In addition to the vertical radiation pattern of the proposed MSSR antenna, Figure 7 illustrates an optimum vertical pattern (the magenta curve) which exhibits a cosec^2^φ shape and has no secondary lobes below the radar horizon. This shape was referenced in various sources and was considered appropriate for PSR and MSSR. The mathematical reasoning behind the optimum vertical pattern is described below.

The Friis transmission formula [4] is written in (24):

(24) $$\frac{P_{rx}}{P_{tx}}=G_{tx}G_{rx}{\left(\frac{\lambda }{4\cdot \pi \cdot d}\right)}^{2}$$

where *P_tx_* is MSSR transmission power and *P_rx_* is the power received by the AVO, *G_tx_* is the MSSR antenna gain, *G_rx_* is the transponder antenna gain, and *d* is the distance between the MSSR and the AVO.

Usually, the gain of the transponder antenna (i.e., λ/4 stub type) is canceled by losses on the feeder and antenna switch, which alternates between the two antennas located on and under the body of the aircraft.

The slant distance in Figure 3 is expressed in (25), considering the right triangle formed by the antenna focus, the aircraft position, and its horizontal projection, where *h* is the reported flight level.

(25) $$d=\frac{h}{\sin\;\varphi }$$

Considering a constant flight level, to ensure a constant reception level at the transponder antenna regardless of the distance variation (or sin φ), the gain of the MSSR antenna *G_Tx_* (26), must be correlated with the square of the slant distance.

(26) $$G_{tx}\approx {\left(\frac{1}{\sin\;\varphi }\right)}^{2}={\cos\mathrm{ec}}^{2}\;\varphi$$

The radiation pattern of the proposed antenna tries to closely replicate the optimum pattern. As shown in Figure 7, the vertical pattern exhibits a wide main lobe which allows it to detect aircrafts at high elevations. Moreover, there was a reduced number of secondary lobes below the 0° elevation, and the SLL was approximately −22 dB. This ensured that the reflections reaching the MSSR weighed less in signal processing—the ultimate goal in MSSR antenna manufacturing. 

The MSSR equipment utilized the Gain-Time-Control (GTC) and Sensitivity-Time-Control (STC) functions, which apply corrections to the received signal level based on the distance between the MSSR and the AVO. The GTC function is a logarithmic attenuation depending on the MSSR–AVO distance in the SUM receiver. STC is a variable sensitivity threshold depending on the MSSR–AVO distance during the time that the GTC is maximum, applied in MSSR extractor. STC is not necessary to be applied for the Roll-Call AVO responses for MSSR Mode S.

### 2.4. 3D Pattern of the MSSR Antenna

The spatial pattern of the MSSR antenna was obtained by performing the product of the horizontal and vertical patterns whose expressions were presented above. Expression |OF·OD|^2^ · |VF·VD|^2^ was written as gain (relative to the maximum for ψ = 0 and φ = 0) in (27).

(27) $$\begin{array}{cc} G_{space\left(\psi ,\varphi )(dB\right)}= & 20\\ & \cdot \log|\sin\left(1.57\cdot \cos\psi \right)\cdot (1.64+2\cdot 1.63\\ & \cdot \cos\left(1\cdot 5.2\cdot \sin\psi \right)+2\cdot 1.60\\ & \cdot \cos\left(2\cdot 5.2\cdot \sin\psi \right)+2\cdot 1.55\cdot \cos\left(3\cdot 5.2\cdot \sin\psi \right)+2\cdot 1.48\cdot \cos\left(4\cdot 5.2\cdot \sin\psi \right)+2\cdot 1.4\\ & \cdot \cos\left(5\cdot 5.2\cdot \sin\psi \right)+2\cdot 1.31\cdot \cos\left(6\cdot 5.2\cdot \sin\psi \right)+2\cdot 1.2\cdot \cos\left(7\cdot 5.2\cdot \sin\psi \right)+2\cdot 1.08\\ & \cdot \cos\left(8\cdot 5.2\cdot \sin\psi \right)+2\cdot 0.97\cdot \cos\left(9\cdot 5.2\cdot \sin\psi \right)+2\cdot 0.85\cdot \cos\left(10\cdot 5.2\cdot \sin\psi \right)+2\cdot 0.73\\ & \cdot \cos\left(11\cdot 5.2\cdot \sin\psi \right)+2\cdot 0.62\cdot \cos\left(12\cdot 5.2\cdot \sin\psi \right)+2\cdot 0.51\cdot \cos\left(13\cdot 5.2\cdot \sin\psi \right)+2\cdot 0.40\\ & \cdot \cos\left(14\cdot 5.2\cdot \sin\psi \right)+2\cdot 0.34\cdot \cos\left(15\cdot 5.2\cdot \sin\psi \right)+2\cdot 0.33\cdot \cos\left(16\cdot 5.2\cdot \sin\psi \right)+2\cdot 0.33\\ & \cdot \text{cos}(17\cdot 5.2\sin\psi )|\\ & \cdot |(\left(\text{cos}\left(1.245\cdot \text{sin}\varphi -0.32\right)\cdot \text{sin}\left(1.57\cdot \text{cos}\varphi \right)/\text{cos}\varphi \right)\cdot (1+2\cdot 0.73\\ & \cdot \cos\left(1\cdot 3.24\cdot \sin\varphi +0.86\right)+2\cdot 0.39\cdot \cos\left(2\cdot 3.24\cdot \sin\varphi +1.14\right)+2\cdot 0.36\\ & \cdot \cos\left(3\cdot 3.24\cdot \sin\varphi +1.34\right)+2\cdot 0.2\cdot \cos\left(4\cdot 3.24\cdot \sin\varphi +1.82\right)+2\cdot 0.13\\ & \cdot \cos\left(5\cdot 3.24\cdot \sin\varphi +1.47\right))|-20\cdot \text{log}101 \end{array}$$

**Figure 8:**
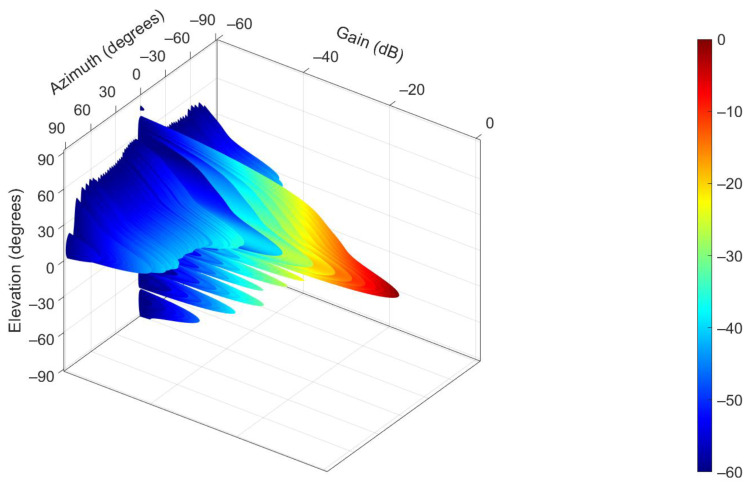
3D pattern of proposed MSSR antenna at 1060 MHz.

The resulting 3D gain pattern is illustrated in Figure 8.

The plot in Figure 8 showcases a main beam that is narrow horizontally and wide vertically. This beam performs 360° clockwise horizontal rotations, transmitting or receiving the MSSR interrogation and response signals, adjusted to the intended coverage. The reflector in this case is considered an infinite perfect conductor. 

**Figure 9:**
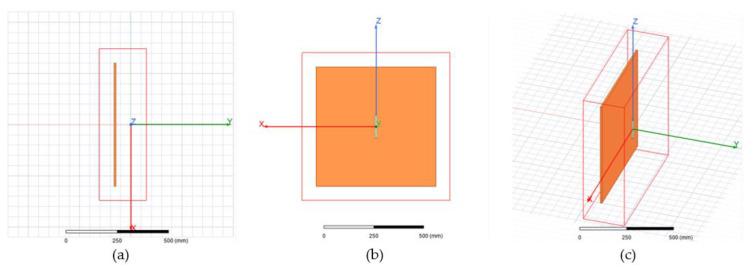
Dipole with panel reflector—single cell of the array: (**a**) top view, (**b**) front view, (**c**) trimetric view.

To evaluate the radiation pattern in a more realistic scenario, a batch of simulations was run in ANSYS HFSS [29], a 3D electromagnetic simulation tool suitable for designing and simulating antennas. HFSS allows the user to generate a composite far-field radiation pattern of a full array, based on a single cell of the array, considerably reducing the computational power. As such, we designed a dipole and the corresponding reflector, and then assigned the array properties (i.e., array dimensions and phase and amplitude of each element). The dipole is illustrated in green in Figure 9, next to the reflector depicted in orange. The reflector for the single cell simulation was 600 mm × 600 mm, which was deemed wide enough, considering the wavelength at 1060 MHz. Once the single-cell simulation was done, the composite far-field radiation pattern was generated for the array, consisting of 35 columns of 11 dipoles each, spaced according to the geometry in Figure 3 and Figure 6. The amplitudes and phases of the feeding currents were computed based on the data in Table 1 and Table 2. 

**Figure 10:**
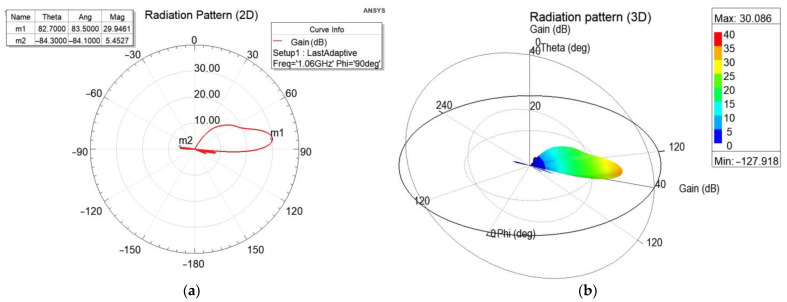
Radiation pattern of the array antenna with panel reflector: (**a**) 2D polar plot, (**b**) 3D polar plot.

The resulting radiation pattern for the array based on the cell in Figure 9 is shown in Figure 10. The main lobe had a gain of approximately 30 dB, while the gain of the backlobe was 5.5 dB. In the MSSR antenna, the backlobe was suppressed by the back column (depicted in Figure 1). However, this aspect was not in the scope of our study. 

## 3. Radar coverage

This section presents main aspects that need to be considered when computing the coverage of radar equipment.

### 3.1. Refraction in the Earth’s Atmosphere

As presented in [3,30,31], to compensate for the phenomenon of refraction in the Earth’s atmosphere, the curvature of the Earth was redrawn by replacing the real form with the theoretical form used in calculations related to the propagation of RF signals. The correction as introduced to consider the rectilinear propagation of the signal. This correction as applied to the Earth’s radius and as introduced by the propagation factor k = 1.3333(3) under normal atmospheric conditions, and for 1060 MHz frequency. 

**Figure 11:**
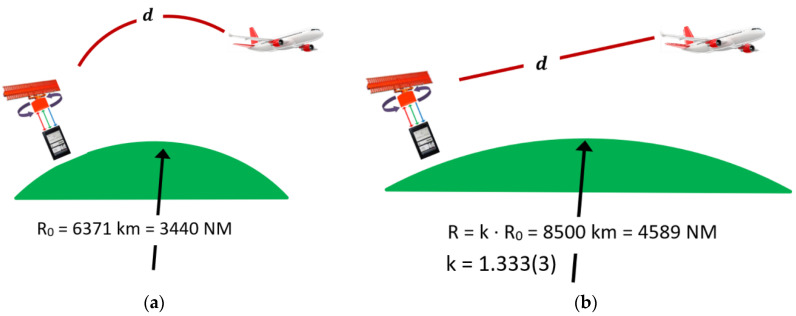
Realistic (**a**) vs. theoretical (**b**) signal propagation in Earth’s atmosphere.

Figure 11 shows this transition, highlighting a theoretical Earth radius of 4589 NM when the signal rectilinear propagation was considered. The length of the physical path traveled by the RF signal remained the same in the two cases, marked *d* in Figure 1 and Figure 11. This paper considered the Earth radius R = 4589 NM.

### 3.2. Flight at Constant Height

**Figure 12:**
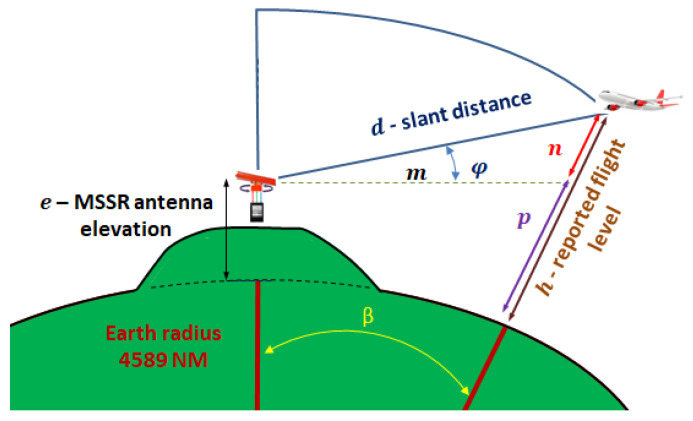
Flight at a constant level, *h*.

Figure 12 illustrates the MSSR equipment and the aircraft at a constant flight level, *h*. The radar extractor computed the slant distance, *d*, and then determined the flight level reported by the AVO transponder, knowing the MSSR antenna elevation and the horizontal projection of the slant distance. The data (i.e., slant distance and flight level) as then sent to the surveillance signal users using the All Purpose Structured Eurocontrol Surveillance Information Exchange (ASTERIX) standard category—CAT1 for MSSR and ASTRIX CAT48 for MSSR and MSSR Mode S. The distances were expressed in NM and flight level in feet, as required by aviation operation standards.

For the triangle formed by the center of the Earth, the MSSR antenna focus and the AVO position:

(28) $${\left(R+h\right)}^{2}={\left(R+e\right)}^{2}+d^{2}-2\left(R+e\right)d\cdot \cos\left(\frac{\pi }{2}+\varphi \right)$$

which can be used to determine the angle *φ* in terms of the slant distance *d* and flight level *h*:

(29) $$\varphi =\arcsin\left(\frac{{\left(R+h\right)}^{2}-d^{2}-{\left(R+e\right)}^{2}}{2\;d\;\left(R+e\right)}\right)$$

Other helpful expressions are:

(30) m=(R+e) tanβ

(31) $$d^{2}={\left(R+e\right)}^{2}+{\left(R+h\right)}^{2}-2\left(R+e\right)\left(R+h\right)\cos\beta$$

resulting in:

(32) $$m=\left(R+e\right)\cdot \tan\left(\arccos\frac{{\left(R+e\right)}^{2}+{\left(R+h\right)}^{2}-d^{2}}{2\cdot \left(R+e\right)\cdot \left(R+h\right)}\right)$$

But for the triangle with the edges *d*, *m*, and *n*:

(33) $$n^{2}=m^{2}+d^{2}-2\cdot m\cdot d\cdot \cos\left(\frac{\pi }{2}+\varphi \right)$$

Generating the flight height *n*:

(34) $$n={\left(d^{2}+(\left(R+e\right)\cdot \tan{\left(\arccos\frac{{\left(R+e\right)}^{2}+{\left(R+h\right)}^{2}-d^{2}}{2\cdot \left(R+e\right)\cdot \left(R+h\right)}\right)}^{2}-2d\left(\left(R+e\right)\;\;\;\;\;\;\;\;\cdot \tan\left(\arccos\frac{{\left(R+e\right)}^{2}+{\left(R+h\right)}^{2}-d^{2}}{2\cdot \left(R+e\right)\cdot \left(R+h\right)}\right)\right)\cdot \cos\left(\arcsin\frac{{\left(R+h\right)}^{2}-d^{2}-{\left(R+e\right)}^{2}}{2*\;d\;\left(R+e\right)}\right)\right)}^{1/2}$$

**Figure 13:**
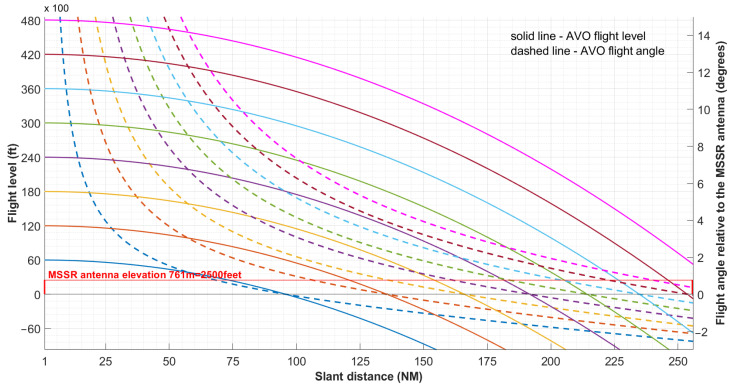
Flight level and flight angle for AVO in flight at constant level as perceived by an observer in the focus of the MSSR antenna (at altitude of 0 ft).

Figure 13 illustrates the variation of the flight level, *n*, and the flight angle of the aircraft as perceived by the MSSR antenna, *φ*, for eight flight levels. 

The plot in Figure 13 shows that there was a lower limit below which the aircraft could no longer be detected because of the curvature of the Earth. For example, an aircraft at a constant flight level of 36,000 ft at a distance of 256 NM from the radar can be seen at −2° below the radar horizon for an MSSR antenna at sea level. If we consider an MSSR antenna at 761 m above sea level (indicated with the red line in Figure 13), an aircraft at a constant flight level of 36,000 ft and at a distance of 256 NM from the radar can be seen at −2.8° below the considered radar horizon. If required to detect targets below the radar horizon (e.g., ensure surveillance for areas close to airports), the antenna must be tilted to rotate the radiation pattern in the vertical plane. 

Figure 13 also shows that the radar coverage was affected by parameters independent from the MSSR transmission/reception capabilities.

Given these limitations, the radar equipment manufacturers state the coverage limit at 256 NM horizontally and 66,000 ft vertically [19,32,33].

### 3.3. Radar Range

Based on (22), (35) expressed the free space pathloss on a logarithmic scale, considering *G_tx_ = G_rx_* = 1:

(35) $$A_{t}=20\log\left(\frac{4\pi d}{\lambda }\right)$$

For the frequency expressed in MHz, and the distance in km, the free space pathloss becomes (36):

(36) $$A_{t}=32.44+20\log f+20\log d$$

Next, the transmission and reception range of the MSSR radar was determined, considering L as additional loss in the system

(37) $$P_{rx}=P_{tx}+G_{tx}+G_{rx}-A_{t}-L$$

(38) $$20\log d=P_{tx}+G_{tx}+G_{rx}-P_{rx}-L-20\log f-32.44$$

(39) $$d=10^{\frac{P_{tx}+G_{tx}+G_{rx}-P_{rx}-L-20\cdot f-32.44}{20}}\;\;\left(\mathrm{km}\right)$$

For radar transmission, we considered the operating frequency of 1030 MHz, the pulse transmit power of 63 dBm, the radar antenna gain of 30 dB (estimated for the proposed MSSR antenna), the cumulative attenuation on the transmit signal path (30 m feeder length, rotary joint, radome) of 8 dB, and the aircraft receiver sensitivity of −72 dBm. An additional attenuation of 3 dB was assumed to be inserted by the aircraft RF circuit. As such, the MSSR-to-AVO maximum range is (40):

(40) $$d_{Tx\_max}=1160\;\mathrm{km}=626\;\mathrm{NM}$$

For radar reception, the operating frequency is 1090 MHz, the transponder transmit power is 54 dBm (respecting the limits indicated in [5]), the aircraft RF circuit attenuation is 3 dB, the radar antenna gain is 30 dB, the cumulative attenuation on the receive signal path (30 m feeds, rotary junction, radome) is 8 dB, and the sensitivity of the SUM reception system is −86 dBm [5]. As such, the AVO-to-MSSR maximum range is (41):

(41) $$d_{Rx\_max}=1950\;\mathrm{km}=1053\;\mathrm{NM}$$

These results show a maximum operation range of 626 NM, computed for the maximum MSSR antenna gain.

The plot in Figure 13 further diminishes the range to 256 NM due to obstruction of low altitude targets and loss of line-of-sight (LoS).

### 3.4. Volume Coverage

**Figure 14:**
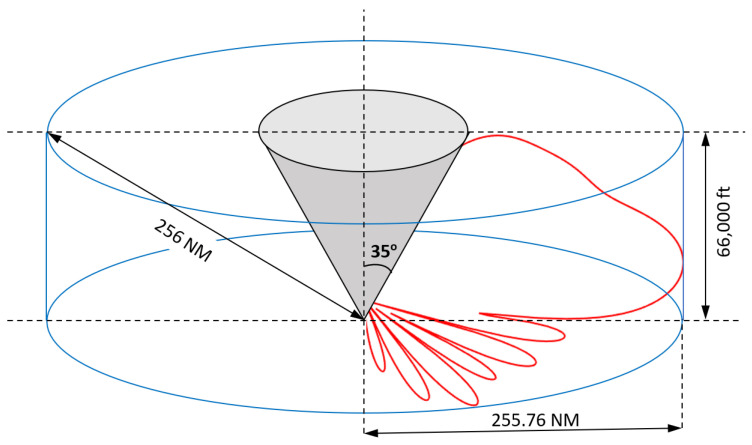
Coverage volume of the MSSR antenna.

Figure 14 illustrates the volume coverage of the MSSR radar. The zenith cone of silence is highlighted. It is a result of using dipole radiant elements in the MSSR antenna and transponder antenna. Increasing the tilt of the radar antenna will decrease the zenith cone but will lose sight of aircrafts flying at a low altitude. Decreasing the tilt of the radar antenna will ensure LoS with low altitude aircrafts but the proper positioning of the AVO on the radar will be degraded by neighboring reflector surfaces (e.g., ground and buildings). To ensure surveillance of wide areas, several radars are deployed with overlapping coverages [34].

### 3.5. Reflections Due to Antenna Positioning

When an electromagnetic (EM) wave meets an environment with a different refractive index, it is affected by the following phenomena: reflection, refraction, diffraction, and dispersion. If the EM wave encounters an obstacle whose dimensions are comparable to the wavelength λ, it will be subjected to diffraction (scattering). If the irregularities of the encountered obstacle are much smaller than the wavelength, it will experience diffusion. The necessary condition for the occurrence of the reflection phenomenon is that when the EM wave propagates in medium 1 and meets medium 2 (e.g., natural obstacles, surfaces covered by ice or snow, soil, soil covered by vegetation, forests, metal roof materials, ceramic or plexiglass, buildings) the size of the surface on which the reflection occurs must be much larger than the wavelength. Otherwise, the occurring phenomenon will be diffusion. If medium 2 is not perfectly conductive, part of the EM signal will enter medium 2, resulting in the refraction phenomenon. The energy of the reflected EM wave is identical to that of the incident EM wave only if the reflection surface is perfectly conductive (as in the case of metals). This situation does not exist in practice. When a surface is less conductive, some of the incident wave energy is refracted or lost in the incident medium; the reflected wave energy is reduced accordingly. The incident electromagnetic wave energy is the sum of the reflected and refracted wave energies according to the conservation principle.

**Figure 15:**
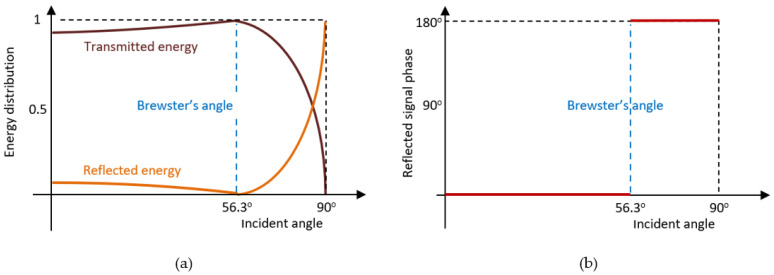
Vertical plane reflection.: (**a**) energy distribution, (**b**) reflected signal phase variation.

In the case of vertical polarization, there is an incidence angle, called the Brewster angle, at which the phase of the reflected signal is reversed, and the entire energy of the incident wave is transmitted to the second medium. As the incident angle increases above the Brewster angle, the energy of the reflected wave increases rapidly, and the phase remains reversed. This aspect is illustrated in Figure 15.

In the case of MSSR radar equipment, the polarization of the transmitted and received signal is vertical and the reflections occur between medium 1 (air) and medium 2 (other than air and whose refractive index is higher). According to Fresnel equations [35], the energy of the reflected signal is dominant, relative to the energy of the refracted signal for large incidence angles (i.e., when the aircraft is located far away from the MSSR antenna).

Figure 15 illustrates signal reflection at the limit between air and fresh snow (which has a refraction index of 1.5 (according to [2,36,37,38]) and analyzed for air-soil in general in [39]). In this case, the Brewster angle was 56.3°, and greatly impacted the amplitude and phase of the signal. 

### 3.6. Multipath Propagation in the MSSR-Aircraft Transmission

The algorithms implemented for reflection mitigation in MSSR equipment lead, in most cases, to the removal of erroneous positions of aircraft. The logic that the direct response from the aircraft transponder always arrives before the reflected response is a method implemented in the MSSR extractor for removing unwanted responses.

However, if the reflecting surface is located very close to the axis of the antenna, the mentioned techniques cannot distinguish the correct response of the aircraft. Figure 1 shows that the duration of a pulse forming aircraft responses is 450 ns for an A–C response (for Mode S transponder response, the duration of the 4 preamble pulses is 500 ns). Thus, if the time difference between the direct response and the reflected response is less than 450 ns (corresponding to a path difference of 135 m), the MSSR extractor will not be able to make a correct assessment of the reception moment, without considering the amplitude of the combined response.

The worst-case scenario of a horizontal reflector near the MSSR equipment is briefly presented below.

**Figure 16:**
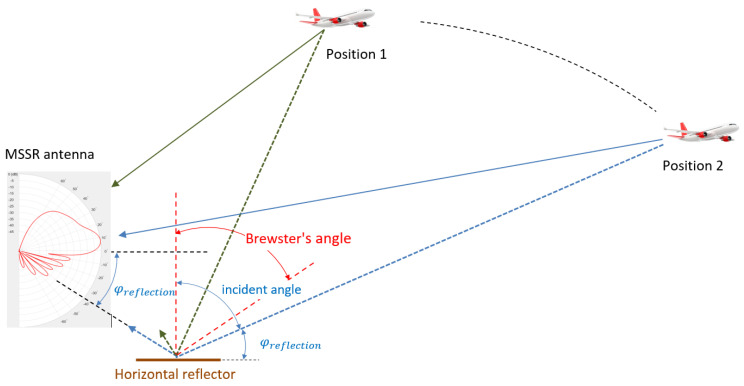
Transponder response reflected by a horizontal surface.

Figure 16 illustrates the multipath vertical reception, similar to [40]. The vertical radiation pattern of the proposed MSSR antenna on 1060 MHz was represented in polar coordinates to showcase the orientation of the main and side lobes. The aircraft was presented in flight at a constant level in two flight positions. A horizontal reflector surface (considered to be the ground covered with fresh snow) was also included. The aircraft response to MSSR queries reached the MSSR antenna on two paths: the direct LoS one (solid line) and the reflected one (dashed line). As highlighted in Figure 16, the angle of incidence was complementary to the angle of signal reception (φ_reflection_).

Considering the energy distribution in Figure 15, when the aircraft was in Position 1, the corresponding reflected signal weighed less than the one corresponding to the aircraft in Position 2. Moreover, the phase for Position 2 was reversed. Throughout the flight, the MSSR antenna would thus sum up two signals with different phases and amplitudes.

The range and angular position of the aircraft and the resulting reception angle (φ_reflection_) determines signal amplitude maxima and minima, producing alternating constructive and destructive patterns [41]. Therefore, the signal at the output of the MSSR antenna shows the lobing phenomenon, due to the reflection with the ground or other reflective surfaces. For the angles of incidence in which the secondary lobes have maximum values, the distance at which aircrafts can be detected increases due to reflections—but this is not an aid in radar surveillance.

The optimal shape of the vertical pattern described in Figure 7 has no secondary lobes below the horizon, leading to the elimination of most harmful reflected signals.

The position of the MSSR antenna also impacts the amount of received reflected signals. An antenna located on a higher tower causes it to collect a greater number of reflections in the vicinity of the MSSR equipment. As such, within a radius of 500 m from the MSSR equipment, the area should be flat and void of reflectors.

The radiation pattern of the MSSR antenna does not change according to environmental conditions. However, the output signal of the antenna (during signal reception) depends on the multitude of input signals (direct and reflected). Moreover, Brewster’s angle causes the combined direct and reflected signals to undergo an instantaneous amplitude change, due to the phase shift of the reflected signal. As such, the lobing phenomenon in [2,3,35,42,43] is not constant, but instead varies with the angular position of the AVO. Due to the reciprocity theorem, the same phenomena would affect the transmissions as well.

**Figure 17:**
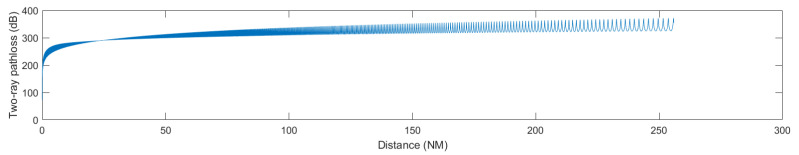
Two-Ray pathloss (transmit antenna height of 2500 ft, receive antenna height of 35,000 ft, ground relative permittivity of 15).

To showcase the variation of the pathloss due to ground reflections, a simple simulation of the Two-Ray pathloss model was performed in MATLAB, using the mathematical apparatus described in [44]. Considering a transmitting antenna height of 2500 ft (i.e., 761 m), receiving antenna height of 35,000 ft (i.e., 10,662 m), a range of 256 NM (i.e., 474 km), and the ground relative permittivity of 15 (suitable for wet ground), the pathloss was calculated, and is shown in Figure 17. Such a variation of the pathloss results in the lobing phenomenon due to reflections.

**Figure 18:**
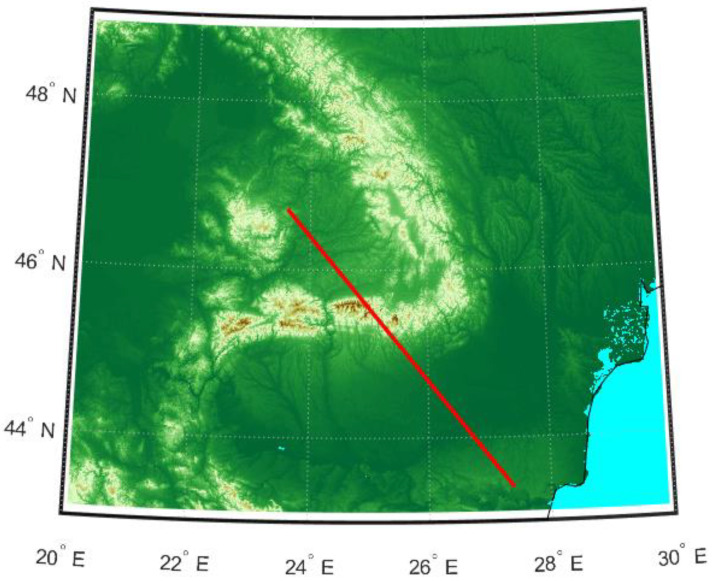
Map of the terrain and path of the analyzed AVO at constant flight level of 35,000 ft.

To showcase the impact of terrain irregularities on the pathloss, a simulation was done using PETool [45], a MATLAB-based parabolic equation tool for radiowave propagation over variable terrain [46]. PETool is able to interpret Digital Terrain Elevation Data (DTED) data from terrain files and plot the pathloss corresponding to a certain path on the map. We selected a map for Romania and set an AVO path at an altitude of 35,000 ft, starting above the MSSR antenna at (46.718519° N, 23.600730° E), and ranging for 474 km on the azimuth of 139.7°, as shown in red in Figure 18. The transmit antenna was placed at an altitude of 2500 ft.

PETool considers a Gaussian antenna pattern when computing the propagation factor and the pathloss by means of the parabolic equations [46]. The antenna type cannot be modified, but the user can set the beamwidth and elevation. Considering the vertical radiation pattern of the proposed MSSR antenna, we set a beamwidth of 12° and an elevation of 8° for the MSSR transmission. Standard atmosphere [46] was considered for the refractivity profile.

**Figure 19:**
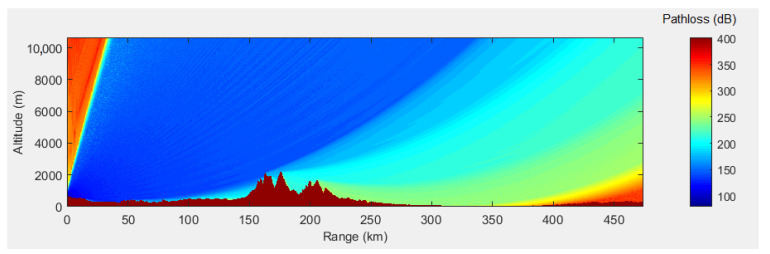
Pathloss variation for the selected path.

The pathloss computed in PETool for the selected path is illustrated in Figure 19. This color-coded plot shows sudden pathloss variations, noticeable especially in the 50–300 km range, where the pathloss rapidly and frequently varies from darker blue to lighter blue, thus exhibiting the lobing phenomenon, due to reflections. 

**Figure 20:**
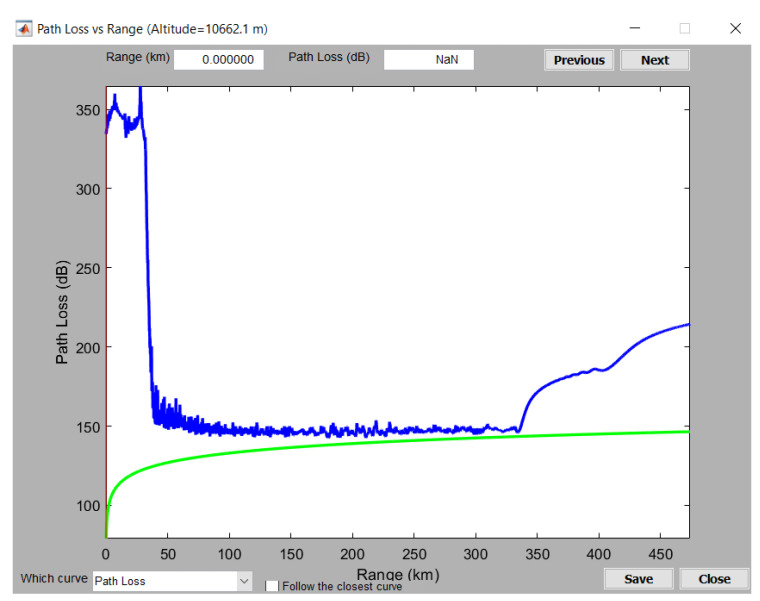
Pathloss variation for the selected path at flight level of 35,000 ft.

For the altitude of 35,000 ft, the pathloss is plotted in Figure 20. The green line represents the free space pathloss, while the blue line shows the overall estimated pathloss, considering the irregular terrain and occurring reflections. Given the configured antenna characteristics, the resulting pathloss exhibits a cone of silence above the antenna location, where the pathloss reaches 350 dB in the range of 0–50 km for the selected flight level (also shown in Figure 19). A similar behavior (i.e., cone of silence above the MSSR) was obtained for the MSSR antenna, as illustrated in Figure 14. 

The PETool simulation results showed the impact of the terrain on the pathloss and, therefore, on the received signal level in the system. Correlating the plots in Figure 19 and Figure 20 shows that, for the altitude of 35,000 ft ,sudden pathloss variations were detected in the range of 50–300 km, generated by terrain irregularities close to the ground antenna.

Although the MATLAB PETool simulations did not take into account the radiation pattern of the MSSR antenna, they clearly showed the major impact that terrain and generated reflections have on radar coverage. Moreover, in a real system, other reflectors might be present (e.g., roofs) that could provoke additional reflections—which are hard to predict.

The output of the MSSR antenna during reception was composed of the direct signal, several reflected signals, signals from other AVOs (the MSSR equipment being able to detect four AVOs on the same azimuth using a degarble algorithm), signals from AVOs that are not in the main lobe field of view (but are interrogated through reflectors in the main lobe FoV and send response signals on the same paths), Automatic Dependent Surveillance–Broadcast (ADS-B) signals, multilateration (MLAT) signals, and signals from AVOs interrogated by other MSSR equipment with overlapping coverage. Essentially, the output of the SUM antenna contained the useful signal and a number of interfering signals.

MSSR Mode S equipment solved many issues related to the anomalies presented on the aircraft display through a series of factors, e.g., reducing the radio frequency spectrum load due to the individual dialogue with the aircraft in the coverage area (each aircraft was interrogated only when it was in the main beam FoV after it was subsequently acquired as a result of the UF11 broadcast interrogation) and adjusting the level of the MSSR query signal in Roll-Call.

However, the multipath issue, especially for low-altitude aircraft, is still unresolved. A brief overview of this issue is presented in [47].

## 4. Experimental Measurements and Simulations

This section of the current work aimed to highlight the impact of the radiation pattern and propagation conditions on the MSRR positioning performance. 

**Figure 21:**
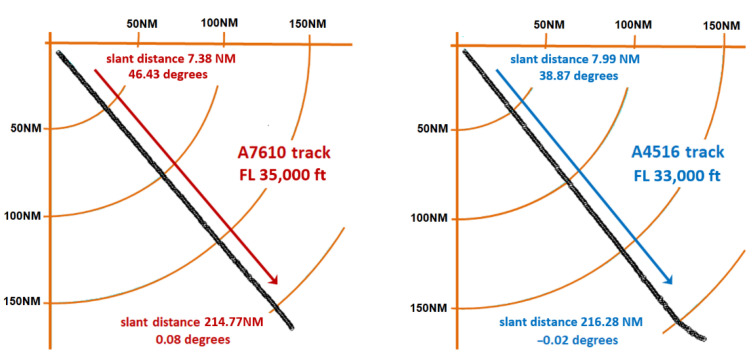
Flight path of A7610 and A4516.

As such, we analyzed two AVOs (i.e., A7610 and A4516) that flew in the coverage area of an MSSR Mode S system in Romania. The AVO tracks are plotted in Figure 21. The AVO position was determined every 8 s, according to the rotational movement of the antenna.

According to the geographical position of the MSSR equipment, the A7610 aircraft flew on azimuth 139.5° and the slant distance calculated in the MSSR extractor was in the range of 7.38 NM–214.77 NM for a flight level of 35,000 ft, while A4516 flew on azimuth 141.5° and the slant distance calculated in the MSSR extractor was in the range of 7.99 NM–216.28 NM for a flight level of 33,000 ft.

**Figure 22:**
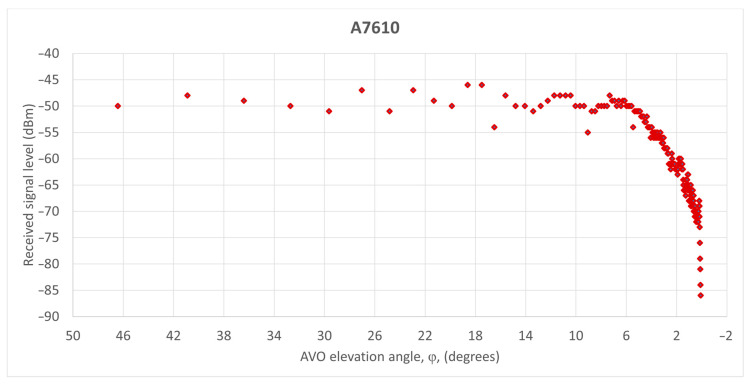
Received signal level variation for A7610.

**Figure 23:**
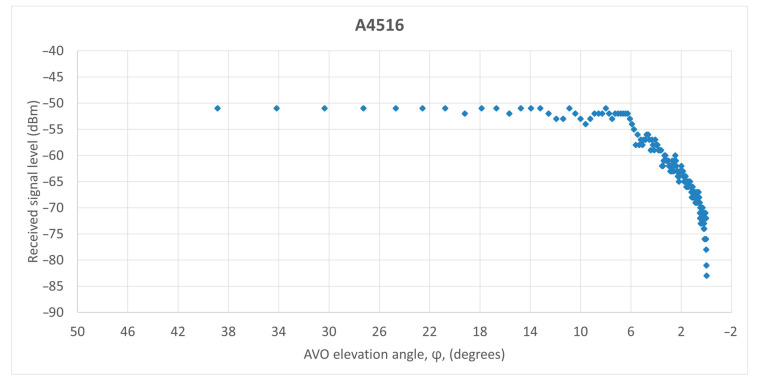
Received signal level variation for A4516.

The signal level detected at the input of the SUM receiver of the MSSR equipment, averaged for the approximately 14 responses per antenna scan (as described in Figure 1) and presented in the structure of ASTERIX CAT48 messages was processed and, according to relation (29), the AVO elevation angle (φ) was calculated for each generated response of the extractor. The graphical representations of the received signal levels according to AVO elevation angle (φ) are shown in Figure 22 (for A7610) and Figure 23 (for A4516). 

The variation of the received signal level with the aircraft elevation angle illustrated in Figure 22 and Figure 23 is as accurate as possible. As the AVO moved away from the MSSR equipment, whose antenna was at 2500 ft elevation (according to Figure 12 and Figure 13), the detected AVO angle (φ) decreased, as expected. However, the detected signal level no longer respected the smooth shape of the cosec^2^ φ section of the vertical pattern. For AVO flight angles above 6°, the signal level exhibited sudden jumps of up to 7 dB that h no other explanation than the combination, during MSSR reception, of the direct signal with signals reflected by geographical or artificial surfaces, as presented in [3]. These figures also illustrated that, at high AVO elevations, where the AVO is close to the MSSR, the reception level is almost constant. This was caused by the GTC function which was applied for a reception window of 309 μs (the equivalent of 50 NM) and resulted in a constant reception level for this range.

Considering AVO A7610 at a flight level of 35,000 ft and an MSSR system equipped with the proposed antenna, a coverage simulation was performed in Radio Mobile. Radio Mobile [48] is a free radio propagation simulation tool based on the ITS Irregular Terrain Model (Longley-Rice) propagation model. It was deemed suitable for the pathloss prediction of our system [49]. The usefulness and accuracy of Radio Mobile in such scenarios was highlighted in [50,51].

The relief map used in the Radio Mobile simulation is DTED1, approximating the real shape of the relief in surfaces whose elevation was defined for units measuring 90 m × 90 m. Compared to the wavelength of the analyzed RF signal (i.e., at 1060 MHz), these dimensions were much larger, resulting in signal reflections.

**Figure 24:**
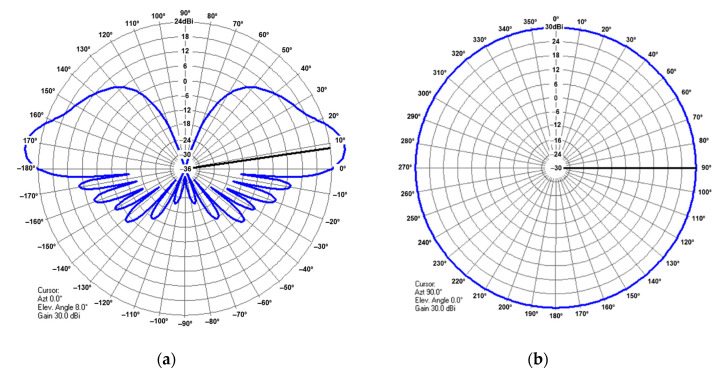
MSSR antenna pattern in Radio Mobile: vertical pattern (**a**), horizontal pattern (**b**).

The simulation scenarios employed the proposed MSSR antenna, defined through the SUM beam pattern for the radar side and a λ/4 stub-antenna for the transponder on board the AVO. Both antennas were introduced as ant files in the simulator. The vertical pattern of the MSSR antenna is shown in Figure 7. To mimic the rotation of the MSSR antenna, the horizontal pattern was considered to be omnidirectional with a gain of 30 dB (as the maximum in Figure 5). The pattern of the MSSR antenna as seen in Radio Mobile is illustrated in Figure 24.

To simulate the operation of the system that provided the results regarding A7610 in Figure 22, we considered the AVO to be the transmitter and the MSSR to be the receiver. The key configuration parameters of the simulation scenario in Radio Mobile are included in Table 3.

**Figure 25:**
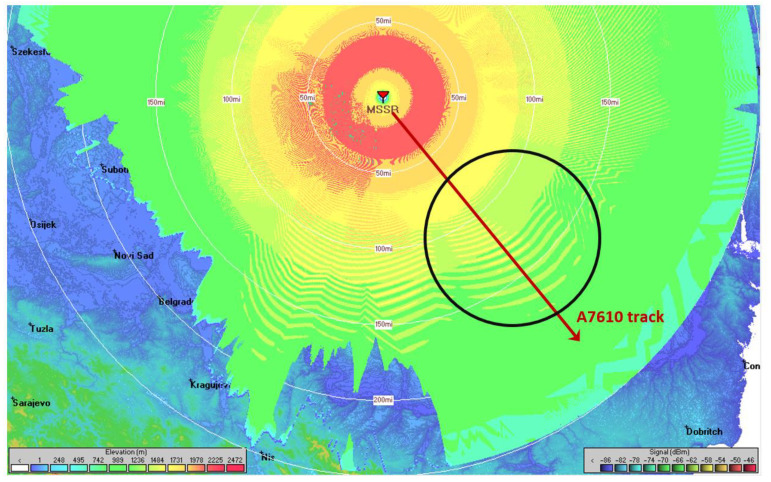
Radio Mobile analysis for AVO A7610 and the 256 NM propagation limit.

The simulation results in Figure 25 showed the lobing phenomenon occurring on the track of AVO A7610 due to reflections caused by the terrain. The black circle on the AVO trajectory in Figure 24 highlights sudden amplitude variations caused by reflections. The pathloss varied faster closer to the MSSR antenna and slower near the end of the 256 NM range, similar to the two-ray pathloss plot in Figure 17 and PETool simulation results in Figure 19 and Figure 20. These results proved that the signal variations in Figure 22 could be caused by ground-induced multipath signal propagation. The reliability of these findings was confirmed by [52].

## 5. Conclusions

This paper analyzed the factors that play a decisive role in the coverage of a monopulse secondary surveillance radar: the antenna radiation pattern, the curvature of the Earth, signal refraction, the link budget based on the capabilities of the MSSR and AVO systems, the reflections that lead to the lobing effect, and the distinctive cone of silence. This paper presented the impact of each of these factors.

First, the radiation pattern of the MSSR SUM beam was assessed, presenting the mathematical apparatus and resulting horizontal, vertical, and 3D patterns for a proposed MSSR antenna. The SUM beam horizontal radiation pattern exhibited a HPBW of 2.41° and a side-lobe level (SLL) of −34 dB which accounted for its very good performance in the determination of the AVO azimuth. Moreover, the SUM beam vertical pattern exhibited only a few side lobes below the radar horizon, with an SLL of −22 dB—which meant that the weight of the reflected signals was reduced. The ideal vertical radiation pattern was also illustrated in this paper, highlighting the cosecant shape of the main lobe.

The radiation patterns presented here are theoretical. Cumulative errors in the manufacturing of the antenna (e.g., errors in cutting the feeders, resulting in phase differences, impedance mismatches, inductive coupling between dipoles, imperfect reflectors, etc.) could lead to antenna performance degradation. However, as illustrated in the paper, the major undesirable changes in the presented patterns are generated by reflections close to the MSSR equipment—resulting in the so-called lobing effect.

The impact of the curvature of the Earth on the MSSR range was illustrated by means of a comprehensive plot (Figure 13) that showed the variation of the perceived AVO flight angle with the slant distance for a given constant flight level. The plot showed that an aircraft with a flight level of 43,000 ft was seen close to the radar horizon at a slant distance of 256 NM, explaining altogether the range limitation of most MSSR equipment.

To support these theoretical findings, the paper also presented MSSR measurement data for two AVO flights. The plots of the received signal level variation showed sudden amplitude changes, induced by reflections, and illustrated that the MSSR range was limited by the curvature of the Earth.

The paper also presented coverage simulations in Radio Mobile for the proposed MSSR antenna and an AVO. The simulations showcased the lobing phenomenon that occurs in radar positioning.

Future work will further address the reflections effect on the radiation pattern, with the goal of contributing to combating reflections in MSSR–aircraft dialogue.

## Figures and Tables

**Table 1:** Currents for the Vertical Columns of SUM Beam.

**Column y**	0	±1	±2	±3	±4	±5	±6	±7	±8
**Current I(y)**	1.64	1.63	1.60	1.55	1.48	1.40	1.31	1.20	1.08
**Column y**	±9	±10	±11	±12	±13	±14	±15	±16	±17
**Current I(y)**	0.97	0.85	0.73	0.62	0.51	0.40	0.34	0.33	0.33

**Table 2:** Currents and Phase Difference Distribution in the Vertical Column.

**x**	0	1	2	3	4	5	6	7	8	9	10
**I(dx)**	0.13	0.2	0.36	0.39	0.73	1	0.73	0.39	0.36	0.2	0.13
**φ_x_(rad)**	1.47	1.82	1.34	1.14	0.86	0	−0.86	−1.14	−1.34	−1.82	−1.47

**Table 3:** Main parameters configuration in Radio Mobile.

Parameter	Value/Setting
Centre position (MSSR antenna)	46.71852° N, 23.60073° E
Elevation data source	DTED
Operating frequency	1060 MHz
Relative ground permittivity	15
Antenna polarization	vertical
Climate	Continental temperate
Role of MSSR	Master
Role of AVO	Slave
MSSR antenna height	20 m
AVO antenna height	10,662 m
AVO transmit power	54 dBm
MSSR sensitivity	−86 dBm
